# Relationship of cognition and structured grocery shopping in older adults with and without mild cognitive impairment

**DOI:** 10.1002/alz70856_107505

**Published:** 2026-01-09

**Authors:** Keith R Cole, Katherine Gifford, Leslie Davidson

**Affiliations:** ^1^ Vanderbilt University Medical Center, Nashville, TN, USA; ^2^ Boston University Chobanian & Avedisian School of Medicine, Boston, MA, USA; ^3^ The George Washington University, Washington, DC, USA

## Abstract

**Background:**

Gait speed may decline years before diagnosis of cognitive impairment. Dual cognitive and gait tasks may enhance gait deficits, but less is known regarding naturalistic dual‐tasks during instrumental activities of daily living, i.e., grocery shopping, as a marker for cognitive decline. This study related cognition to structured grocery shopping performance using wearable technology in older adults with mild cognitive impairment (MCI) and cognitively unimpaired (CU).

**Methods:**

Participants included 15 in each group of MCI and CU. Cognitive performance was measured with the NIH Toolbox. A simulated naturalistic dual‐task involved 5 structured shopping trials of collecting eight shopping list items in a simulated physical grocery store. Participants wore an action camera and inertial measurement units at the lumbar spine and posterior head. Shopping outcome measures included whole‐trial actigraphy analysis‐based activity counts from the accelerometer, and degrees and degrees per minute of movement from the gyroscope. Linear regression models related independent variables of age, 10m gait, cognitive status, and cognition to shopping outcomes.

**Results:**

Age was similar between groups (*p* = 0.07). MCI had slower accelerations per minute and gyroscope degrees per minute at the head (*p* <0.001, d=0.51; *p* <0.001, d=0.69) and lumbar spine (*p* = 0.003, d=0.0.60; *p* <0.001, d=0.66) compared to CU. Regression models using the Flanker test explained the greatest amount of variance, explaining 26% and 37% at the head (*p*'s <0.001) and 21% and 22% and at the lumbar spine (*p* <0.001) of acceleration and degrees of movement, respectively.

**Conclusions:**

Objective measurements of movement using wearable technology during real‐world activities, such as grocery shopping, may be a digital biomarker of cognitive and physical function. As cognition declines, in particular executive functioning, the overall accelerations and speed of rotational movements particularly at the head may reflect more significant changes in cognition. Future studies are needed in community grocery stores to assess free‐living IADLs to determine if such activities can be used as a digital biomarker of cognitive, motoric, and functional decline.

